# Early Changes in the Plasma Lipidome of People at Very High Cardiovascular Risk: A New Approach to Assessing the Risk of Cardiovascular Changes

**DOI:** 10.3390/biomedicines13030643

**Published:** 2025-03-06

**Authors:** Joanna Waś, Piotr Dobrowolski, Aleksander Prejbisz, Magdalena Niedolistek, Ilona Kowalik, Anna Drohomirecka, Dorota Sokołowska, Jolanta Krzysztoń-Russjan

**Affiliations:** 1 Department of Medical Biology, National Institute of Cardiology, State Research Institute, 42 Alpejska Str., 04-628 Warsaw, Poland; mniedolistek@ikard.pl (M.N.); dsokolowska@ikard.pl (D.S.); jkrzyszton@ikard.pl (J.K.-R.); 2 Department of Epidemiology, Cardiovascular Disease Prevention and Health Promotion, National Institute of Cardiology, State Research Institute, 42 Alpejska Str., 04-628 Warsaw, Poland; pdobrowolski@ikard.pl (P.D.); aprejbisz@ikard.pl (A.P.); 3 Clinical Research Support Centre, National Institute of Cardiology, State Research Institute, 42 Alpejska Str., 04-628 Warsaw, Poland; ikowalik@ikard.pl; 4 Heart Failure and Transplantology Clinic, National Institute of Cardiology, State Research Institute, 42 Alpejska Str., 04-628 Warsaw, Poland; adrohomirecka@ikard.pl

**Keywords:** cardiovascular high risk, lipidomics, metabolomics, hypercholesterolemia

## Abstract

**Background/Objectives:** Cardiovascular disease (CVD) remains the leading cause of death worldwide and requires a deeper understanding of its pathogenesis for effective prevention and treatment. Familial hypercholesterolemia (FH), characterized by high levels of LDL cholesterol, is a significant risk factor for CVD. FH background remains unexplained despite advances in genetic testing. The aim was identification early changes in the plasma lipidome of individuals at high cardiovascular risk (HCVR) using liquid chromatography coupled with mass spectrometry. **Methods**: The lipidomic analysis examined over 400 compounds. Twenty individuals with suspected FH, very high cardiovascular risk (VHCVR), and undetectable mutations in the *LDLR*, *APOB*, or *PCSK9* genes were compared to control group in a qualitative-quantitative analysis. **Results**: Multivariate analyses revealed statistically significant alterations in glycerophospholipids (GC), with a notable increase in phosphatidylcholines ((O-36:0/16:0), OR (95% CI): 1.246 (1.042–1.490), *p* = 0.0157), phosphatidylethanolamines ((O-40:7/22:6), OR (95% CI): 1.119 (1.039–1.205), *p* = 0.0028), and phosphatidylglycerol ((40:8/20:4), OR (95% CI): 1.053 (1.008–1.101), *p* = 0.0219) only in patients with HCVR. These changes, particularly in major classes of GC, underscored their potential as biomarkers for early assessment of cardiovascular risk. Lipidomic profiling revealed associations between specific lipid species and the comorbidities of arterial hypertension, atherosclerosis, and insulin resistance, implicating their role in atherosclerotic cardiovascular disease (ASCVD). **Conclusions**: This study points early changes in the plasma lipidome in individuals at HCVR, underline potential biomarkers, therapeutic targets for ASCVD, and offer opportunities to improve ASCVD diagnosis, therapy, and risk management strategies through detailed personalized medical approach.

## 1. Introduction

Cardiovascular disease (CVD) remains one of the leading causes of morbidity and mortality in both Poland and globally. Among the various risk factors contributing to CVD development, familial hypercholesterolemia (FH) is of particular interest due to its solid genetic basis LDL receptor (LDL-R), apolipoprotein B-100 (APOB), and protein convertase subtilisin/kexin type 9 (PCSK9). Mutations of the genes encoding the above-listed proteins lead to the impaired clearance of low-density lipoprotein (LDL) from the bloodstream, resulting in their accumulation within the arterial walls. FH is characterized by elevated levels of LDL, strongly associated with an increased risk of premature atherosclerosis, coronary artery disease, and other cardiovascular diseases [1,2].

Although molecular diagnostic methods have allowed the identification of these mutations in a significant proportion of patients, there remains a subset of individuals in whom such mutations are detected despite clinical features suggestive of FH. This discrepancy underscores the need for additional biomarkers that can aid in diagnosing and managing FH, particularly in cases where genetic testing is inconclusive [3,4]. Misdiagnosis or delayed diagnosis of FH can lead to severe cardiovascular complications, further highlighting the urgency of developing additional biomarkers [5]. Furthermore, lipidomics can provide insights into the dynamic changes in lipid metabolism that occur in response to therapeutic interventions, helping to develop more effective CVD treatments [5,6,7,8,9,10,11,12,13]. One of the critical benefits of lipidomics is its ability to reveal subtle changes in lipid composition that may not be detectable by conventional lipid measurements, such as total cholesterol or LDL-C levels. For example, specific alterations in the composition of phospholipids, sphingolipids, or other lipid classes may serve as early indicators of disease processes, offering new avenues for early diagnosis and intervention.

The purpose of the present study was to comprehensively analyze the lipidomic profile of people with high cardiovascular risk (HCVR) using advanced mass spectrometry techniques. This study’s findings can inform the development of new biomarkers for FH and other forms of CVD, ultimately contributing to an improved diagnosis, risk stratification, and therapeutic strategies for these conditions.

## 2. Materials and Methods

### 2.1. Study Population

The study population (n = 40), made up of 20 patients (Patient Group (PGr)) (n = 20), consisted equally of 10 women and 10 men with a mean age value of 50.6 (±7.4) and with high total cholesterol and a high LDL fraction (Table 1). All tested patients were diagnosed with HCVR and suspected FH. No pathogenic mutations were found in the examined group’s *LDLR*, *APOB*, or *PCSK9* genes. Very high cardiovascular risk (VHCR) was defined according to the ESC 2021 guidelines [14]. The criteria for inclusion in the study were as follows: (1) CVD, including atherosclerotic cardiovascular disease (ASCVD), and (2) elevated total cholesterol and LDL lipoprotein fraction, without mutations in the *LDLR/APOB/PCSK9* genes. The criteria for exclusion from the study were as follows: (1) lack of informed consent of the patient to the examination; (2) age < 18 years. The Control Group (CGr) (n = 20) consisted of 20 phenotypically and genotypically consecutive volunteers who did not meet the high/very high cardiovascular risk; they were matched by age (mean age: 49.0 ± 10.9), and there were 10 women and 10 men. All patients were diagnosed in the Outpatient Lipid Clinic of the National Institute of Cardiology—State Research Institute in Warsaw, Poland.

The regional Ethics Committee approved the study (approval no. 1894). All patients and volunteers gave their written consent to participate in the study.

### 2.2. Blood Sampling

Venous blood samples derived from the participants were centrifuged (5702R: Eppendorf, Hamburg, Germany), at 2000× *g* for 15 min at room temperature, and the plasma was aliquoted and stored at −80 °C (TSE320VGP: Thermo Scientific, Thermo Fisher Scientific, Bremen, Germany) until analysis. Repeated freeze–thaw cycles were avoided. Lipids were isolated for lipidomics studies using organic solvents: acetonitrile, chloroform (Chempur Silesian Piekary, Piekary Śląskie, Poland) and methanol (J.T. Baker, Avantor, Gliwice, Poland), of LC-MS purity [15,16]. In both groups, biochemical assays were performed, including the concentration of low-density lipoprotein (LDL fraction) and high-density lipoprotein (HDL fraction) and triglyceride content.

### 2.3. Sample Preparation

An aliquot of 75 µL of the experimental plasma samples was transferred to glass culture tubes (3 DRAM, 19x65 ST, Finneran, Vineland, NJ, USA). To all samples, 0.9 mL of water, 2 mL of methanol, and 0.9 mL of dichloromethane (Chempur Silesian Piekary, Poland) were added. The samples were gently vortexed (V-1 plus: Vortex, Biogenet, Warsaw, Poland) for 5 s and then placed on the bench top at RT for 30 min. Next, 1 mL of water and 0.9 mL of dichloromethane were added to the samples. The samples were gently vortexed for 5 s and then centrifuged at 2800× *g* for 10 min at room temperature or until the extracts were visibly separated into a bilayer. Since over-vortexing at this stage could easily result in poor phase partitioning, the bottom of the organic layer was transferred to a new tube for each extract. Another 1.8 mL of dichloromethane was added, and the original samples were gently vortexed for 5 s and centrifuged at 2800× *g* for 10 min at room temperature or until the extracts were visibly separated into a bilayer. The combined bottom layers for each sample were then concentrated under nitrogen and reconstituted in 0.25 mL of the running solution (10 mM ammonium acetate (Merck, Darmstadt, Germany) in a mixture of dichloromethane–methanol (50/50 *v*/*v*). The extracts were then transferred to inserts and placed in vials (J0602: Chromsystems Instruments and Chemicals GmbH, Grafelfing, Germany) for LC-MS analysis.

### 2.4. Lipidomic Analysis

Qualitative lipidomic analyses were performed on more than 400 lipid compounds classified into different classes (cholesterol esters, triglycerides, diglycerides, free fatty acids, bile acids, phosphatidylcholine, phosphatidylethanolamine, phosphatidylinositol, phosphatidylserine, sphingomyelins, and ceramides).

HPLC conditions: An automated flow injection analysis (FIA) was performed using an ExionLC AD HPLC (Sciex, Framingham, MA, USA) set consisting of two pumps in a gradient system and a thermostatic autosampler in the MS/MSALL Infusion mode. The autosampler was connected directly to the ion source via a 100 cm PeekSIL Security LINK capillary with a diameter of 50 µm (Phenomenex, Torrance, CA, USA). The mobile phase used was a mixture of methylene chloride–methanol (50:50 *v*/*v*) with the addition of 5 mM ammonium acetate (LC/MS purity) with isocratic flow. The autosampler needle was washed with 100% isopropyl alcohol to reduce the differences between individual injections (overhead). The total chromatographic analysis time was 12 min. Equilibration was performed at 1.2 min, the data acquisition was performed from 1.2 to 6.5 min (flow rate 7 µL/min), and washing was performed from 8.0 min to 12 min (flow rate 100 µL/min).

MS/MS conditions: Measurements were performed on a SCIEX TripleTOF^®^ 6600+ mass spectrometer with an OptiFlow^®^ source (Sciex, Framingham, MA, USA) connected to a 65 μm diameter electrode. Data were collected in the Infusion MS/MSALL mode, consisting of a TOF MS scan (5 s) and a series of MS/MS scans (300 msec.). The total analysis time, including rinsing and conditioning steps, was 12 min. The selected parameters of the ion source were optimized separately for the results during positive and negative ionization under the following conditions: voltage in the capillary +5000 V and −4500 V, temperature −150 °C, shielding gas at 25, gas 1 at 25, and gas 2 at 30. An amount of 50 µL of solution from each sample was injected and analyzed in the mass range of 400–1200 *m*/*z*, with positive and negative polarization in the first and subsequent injections, respectively. To confirm the reproducibility of the results, each sample was injected six times (three injections in positive polarity and three injections in negative polarity). As a result of the infusion MS/MSALL analysis, fragmentation spectra were obtained for all masses available in the sample.

### 2.5. Statistical Analysis

Due to the small number of individuals, comparisons for continuous variables were made using the nonparametric Mann–Whitney test, and the significance for proportions was verified with Fisher’s exact test. The numerous tests resulted in a type I error inflation (raw *p*-value). To address this issue, we used an adjustment for multiple comparisons. Since the conventional Bonferroni correction is too conservative due to the correlation among the test statistics, we utilized an alternative method based on the bootstrap approach, specifically the False Discovery Rate (FDR), to evaluate the significance of the entire lipogram [17]. However, due to the small sample size, none of the results were statistically significant. Therefore, we only performed the multiple comparison correction on statistically significant variables in a single Mann–Whitney test. Volcano plots show the relationship between the log2-fold change (FC) (the ratio of the medians of the subjects from the Patient and the Control Groups) and -log10 separately for subgroups. Multivariate binary logistic regression was performed to minimize multidimensionality. In the first stage, separate analyses were carried out in the three lipid groups (based on statistically significant variables obtained in the bootstrap method), and then significant variables of these models were combined into one. In each case, the backward variable selection procedure was used. Due to the high lipid concentration values, the odds ratios (95% CI) are presented rescaled (unit 10). Drug therapy was not included in the regression analysis because the purpose was only to identify the most essential lipids from a large number of them. We assume the same therapeutic effect of drugs on each of the tested compounds. Univariate receiver operating characteristic (ROC) curves were constructed to determine the diagnostic power of significantly dysregulated lipids. All hypotheses were two-tailed with a 0.05 type I error. Statistical analyses were performed with SAS version 9.4 (SAS Inc. Institute, Cary, NC, USA).

**Table 1:** Population characteristics and general cholesterol evaluation results of Patient and Control Groups.

Demographic Data *	Patient Group	Control Group	*p*-Values
	n = 20	n = 20	
Age {year}, median (IQR)	51 (46–56)	50 (41–55)	0.520
Male, n (%)	10 (50)	10 (50)	1.00
CA/CAD, n (%) **	8 (40)	0 (0)	0.003
BMI (kg/m^2^), (mean ± SD)	28.3 ± 4.3	26.2 ± 5.1	0.064
High cardiovascular risk evaluation during recruiting **			
High cardiovascular risk, n (%)	12 (60)	0 (0)	<0.001
Very high cardiovascular risk, n (%)	8 (40)	0 (0)	0.003
Hypertension, n (%)	7 (35)	0 (0)	0.008
Hypercholesterolemia, n (%)	10 (50)	0 (0)	<0.001
CKD, n (%)	2 (10)	0 (0)	0.487
CThD, n (%)	1 (5)	0 (0)	1.00
Cholesterol and general fraction evaluation			
Total cholesterol {mg/dL}, median (IQR)	212 (145–285)	190 (172–235)	0.797
HDL {mg/dL}, median (IQR)	53 (43–62)	53 (42–65)	0.914
HDL %, median (IQR)	30.4 (22–38)	278 (20–37)	0.882
Non-HDL {mg/dL}, median (IQR)	143 (53–223)	146 (102–174)	0.968
LDL {mg/dL}, median (IQR)	125 (54–204)	133 (95–162)	0.745
Triglyceride {mg/dL}, median (IQR)	132 (103–189)	116 (89–202)	0.546
Atherogenic index, median (IQR)	3.30 (2.64–4.57)	3.73 (2.69–4.97)	0.882
	Lipid-lowering therapy		
Statin	6 (30)	0 (0)	0.020
PCSK9	5 (25)	0 (0)	0.047

* High cardiovascular risk was defined according to the POL-SCORE chart. Patients with diabetes mellitus and chronic kidney disease were considered to have a high cardiovascular risk. Patients with established cardiovascular disease were classified as having VHCVR [18]. ** CA/CAD—atherosclerosis of the carotid or coronary arteries.

## 3. Results

The clinical and biological characteristics of the patients (PGr) (n = 20) and the volunteers that made up the reference groups (CGr) (n = 20) are presented in Table 1. The groups did not differ in age, sex ratio, total cholesterol, and lipid fractions, including LDL, HDL, HDL-%, non-HDL, triglycerides, and the atherogenicity index.

**Figure 1:**
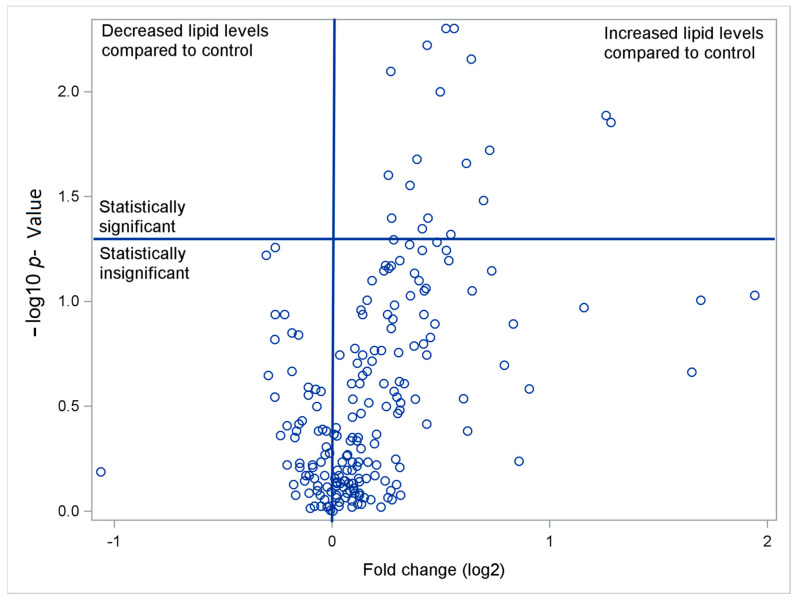
Volcano plots show the FC. To address this issue, we applied an adjustment for multiple comparisons. Since the conventional Bonferroni correction is too conservative due to the correlation among the test statistics, we utilized an alternative method based on the bootstrap approach, specifically the False Discovery Rate (FDR), to evaluate the significance of the entire lipogram of GC from Sg-1. The values of statistically significant GC (above the horizontal line) are listed in Table 2.

**Figure 2:**
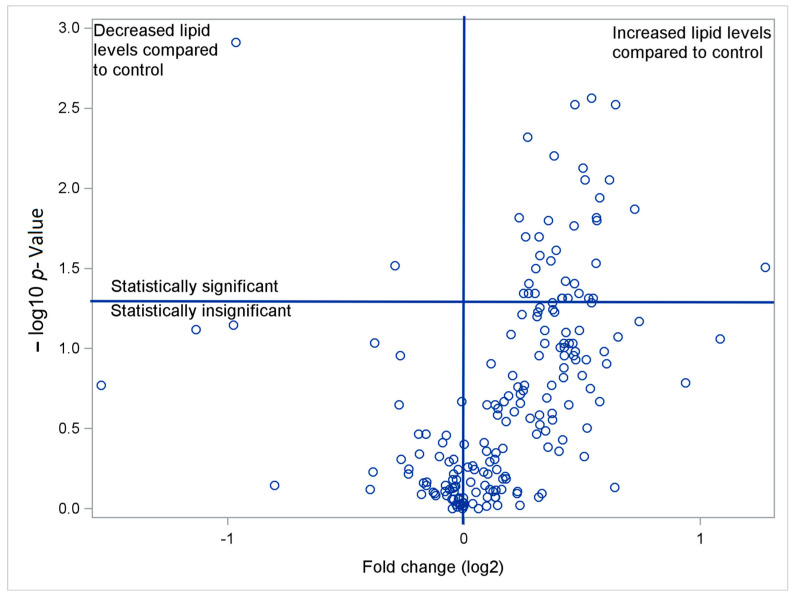
Volcano plots show the FC values of GC from Sg-2. The values of statistically significant GC (above the horizontal line) are listed in Table 3.

When comparing the results of the PGr and CGr, it was shown that out of 222 tested glycerophospholipid components of subgroup-1 (Sg-1), the concentrations of 22 (9.9%) were statistically significantly different (included in the testing were phosphatidylcholine (PC), phosphatidylethanolamine (PE), phosphatidylglycerol (PG), phosphatidylserine (PS)). Out of 201 tested glycerophospholipid components of subgroup-2 (Sg-2), the concentration of 39 (19.4%) was statistically significantly different (included in the testing cardiolipin (CL), monomethyl phosphatidylethanolamine (MMPE), dimethyl phosphatidylethanolamine (DMPE), and diacylglycerol pyrophosphate (DGPP)). Out of 20 sphingolipids tested, 2 (10%) were found to be statistically significantly different. It was not shown that any of the fatty acid components tested differentiated the two groups. For a detailed list of identified lipid compounds, including glycerophospholipids (GC) (Sg-1 and -2), sphingolipids, sterols and acyls, and glycerolipids, see Supplementary File S1. In the CGr, most PC levels were undetectable, while all values were higher in the PGr. Table 2 presents the median {25th percentiles–75th percentiles} levels of statistically significant GC from Sg-1 (PC, PE, PG, and PS) registered in the PGr and CGr. The fold change (FC) remained within the range of 1.21 to 2.43 (Figure 1 and Figure 2). Volcano plots show the relationship between the log2-FC (the ratio of the medians of the subjects from the PGr and the CGr) and -log10 *p*-value.

The results for lysophosphatidylcholine (LysoPC), phosphatidic acid (PA), phosphatidylinositol (PI), phosphatidylinositol phosphate (PIP), and phosphoethanolamine (NAPE) were statistically insignificant. Furthermore, in four instances, these values were undetectable in more than 10 patients in the CGr. Table 3 presents the median {25th percentiles–75th percentiles} levels of statistically significant GC from Sg-2 (Cl, DGPP, MMPE, DMPE, phosphatidylinositol phosphate (PIP), PIP2, PIP3, 3-cytidine diphosphate diacylglycerol (CDPDAG), and phosphoethanolamine (NAPE)) measured in the PGr and CGr. The FC ranged from 0.0 to 2.41.

**Figure 3:**
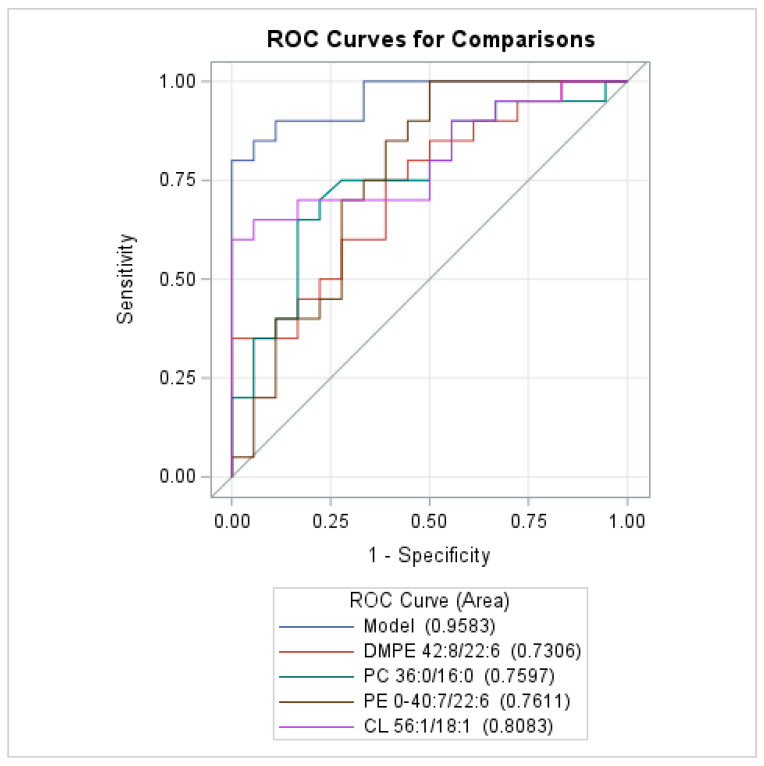
Comparison of the diagnostic power of significantly dysregulated lipids. ROC curve analysis. DMPE—dimethyl phosphatidylethanolamine; PC—phosphatidylcholine; PE—phosphatidylethanolamine; CL—cardiolipin.

Table 4 presents the results of the multivariate analyses based on the significant factors obtained in the univariate analysis of each lipid Sg (Sg-1, -2, and sphingolipids) and the final combined model. In the model based on GC from Sg-1, the discriminant factors were PC (O-36:0/16:0), PE (O-40:7/22:6), and PG 40:8/20:4. In the model based on GC from Sg-2, the discriminant factors were DMPE 42:8/22:6, CL 56:1/18:1, and CL 82:13/18:1. In the final multivariate analysis, only PC (O-36:0/16:0), PE (O-40:7/22:6), DMPE 42:8/22:6, and CL 56:1/18:1 remained independent factors. The discriminatory power of each significant factor is shown in Figure 3.

## 4. Discussion

Innovative research into the pathophysiological mechanisms associated with ASCVD is critical for understanding its pathogenesis and effective prevention and treatment. Identifying risk factors such as hypertension, elevated cholesterol levels, smoking, diabetes, obesity, and genetic risk is crucial. This study focuses on the association between lipid profiles and HCVR, with the aim of exploring potential links to the development of ASCVD and identifying strategies to reduce cardiovascular risk. We can improve patient outcomes by understanding these mechanisms and identifying effective interventions [14,18]. While traditional clinical risk factors are critical for predicting ASCVD, lipidomic profiling offers additional insights for improved risk stratification. Several studies have demonstrated the potential of lipidomics to identify lipid species correlated with ASCVD events, which can improve predictive models. Inconsistencies among studies arose from differing methodologies and lipid species analyzed, emphasizing the need for standardized protocols. The retrospective nature of current studies limits their broad applicability. Prospectively designed studies with diverse populations are needed to validate the association of lipidomic markers with ASCVD and their clinical performance. In the context of FH, lipidomic analysis may differentiate FH patients from those at HCVR due to other factors, revealing unique lipid signatures that may require aggressive therapeutic intervention. However, research that explicitly examines FH lipidomic profiles is limited, indicating a gap that needs to be addressed. Integrating lipidomic data into clinical practice faces challenges due to variations in lipidomic profiles across different studies. Critical steps include identifying lipidomic biomarkers with substantial prognostic value and ensuring that future studies are adequately powered to detect associations [5].

There is a significant gap in our knowledge of the biological pathways associated with an increased risk of cardiovascular disease. Wang et al. analyzed metabolic pathways and provided information on the metabolic implications of differences in lipid profiles. This highlights the need for further analysis of functional and metabolic changes involving specific enzymes to understand better the metabolic processes associated with different etiologies of cardiovascular disease [19]. Several studies have analyzed and compared metabolic pathways of different CVDs with controls, but studies comparing lipidomic pathways of different etiologies need to be improved [4]. Using qualitative lipidomic analysis of more than 400 lipid molecules classified into various classes, lipids have been identified as a factor that differentiates patients at high risk of developing heart disease from healthy subjects [20]. Traditional lipid analysis methods typically focus on a small subset of lipid species, such as cholesterol or triglycerides. In contrast, shotgun lipidomics allows the analysis of hundreds of lipid species in a single experiment, providing a more comprehensive picture of the lipidome, which is essential in disease processes and enables new insights into the role of lipids in health and disease [6,21,22].

Our studies revealed significant differences in glycerophospholipid profiles between patients at high risk for hypercholesterolemia and the Control Group, despite no variation in traditional lipid profiles (i.e., cholesterol, triglycerides (TG), LDL, and HDL). In particular, 22 of the 222 identified GC of Sg-1 (PC, PE, PG, PI, and PS) were statistically significantly different, highlighting their potential role in the pathophysiology related to cardiovascular disease risk. In turn, 39 out of 201 GC of Sg-2 (Cl, DGPP-DG, MMPE, and DMPE) also showed significant differences, although these changes were less pronounced.

Glycerophospholipids play a crucial role in the structure of cell membranes, and their dysfunction may cause disturbances in cell integrity and contribute to the development of atherosclerotic processes [23]. Phosphatidylcholine and phosphatidylethanolamine are the significant components of cell membranes, and their dysfunction can affect membrane permeability and fluidity, promoting lipid accumulation and inducing inflammatory processes in blood vessel walls [11,24,25].

Our studies showed that the components PC (O-36:0/16:0), PE (O-40:7/22:6), and PG 40:8/20:4 were essential factors that differentiated the study groups. We considered the hypothesis that changes in these components may act as markers of early atherosclerotic lesions, but more studies focused on specific pathways are needed [8,26,27,28,29]. Using these biomarkers may allow early identification of patients at increased risk of atherosclerosis and referral for more advanced testing, which may prevent the development of serious cardiovascular complications.

Although PC and PE showed significant differences between high-risk patients and controls, changes in other lipid classes were also substantial. For example, CL (56:1/18:1); CL 82:13/18:1 and DMPE (42:8/22:6), which showed differences between groups, may indicate subtle changes in lipid metabolism that may indicate the early stages of metabolic dysfunction in plasma. These changes may lead to more severe atherosclerosis over time, as early metabolic dysfunctions in lipid handling may set the stage for the progressive development of atherosclerotic plaques. However, it should be noted that not all classes of lipids, such as sphingolipids and fatty acids, showed significant differences in the context of ASCVD, which may indicate that their role in the pathogenesis of atherosclerosis is less critical or that their effects are more subtle compared to those of GC. This suggests that atherosclerosis research should concentrate on more promising lipid classes directly affecting inflammation and cellular dysfunction [30,31,32].

In the context of cholesterol and its fractions, LDL (low-density lipoprotein), HDL (high-density lipoprotein), and non-HDL (all lipoproteins containing apolipoprotein B), their role in the development of atherosclerosis and other cardiovascular diseases should be understood in a broader metabolic context. LDL, the primary cholesterol transporter, can lead to the formation of atherosclerotic plaques when cholesterol accumulates on the walls of blood vessels. HDL, on the other hand, helps to remove excess cholesterol from tissues and transports it to the liver, preventing the development of atherosclerosis. Non-HDL, which is a carrier of apolipoprotein B, may also contribute to the development of atherosclerosis. Ester cholesterol, TG, and DG play essential roles in lipid metabolism, and their disorders may affect the risk of developing atherosclerosis [2,33].

The results of our research show that the analysis of lipid profiles, including cholesterol and glycerophospholipid fractions, can provide important information about the risk of developing cardiovascular disease. Differences in LDL, HDL, non-HDL cholesterol, ester cholesterol, TG, and DG concentrations in different groups of patients may indicate potential metabolic changes associated with the risk of atherosclerosis. Changes in LDL and HDL cholesterol profiles may reflect early stages of atherosclerosis development that have not yet been detected by standard lipid tests. Changes in the profiles of GC, such as PC and PE, may indicate the dysfunction of cell membranes and signaling processes that may be important in the pathogenesis of cardiovascular disease.

The analysis of GC and other classes of lipids can provide new insights into the complex interactions between genes, environment, and metabolism, which is crucial for the development of personalized medicine [20,27]. Despite the promise of lipidomics, there are still issues that need to be addressed to realize its potential in clinical practice fully. Critical considerations include the standardization of analytical methods, the need for large-scale validation studies, and the integration of lipidomic data into existing clinical frameworks. However, the knowledge gained from lipidomics research already contributes to a more nuanced understanding of lipid metabolism and its role in cardiovascular disease [28].

### Limitation of the Study

The study population is relatively limited in size, requiring further research in a larger cohort using predefined lipids. The lack of validation studies and specific pathway analyses should also be addressed. Additionally, the influence of hypolipidemic therapy (statins and PCSK9 inhibitors) on the lipidomic profile cannot be excluded. Lipid-lowering therapy was not included in the multivariate analysis. This limitation should be considered when interpreting the results.

## 5. Conclusions

The findings of this study highlight significant early alterations identified through a comprehensive comparison of lipid profiles between subjects at high risk for cardiovascular events and those in a Control Group.

Specifically, these changes pertain to selected compounds of phosphatidylcholine, phosphatidylethanolamine, phosphatidylglycerol, phosphatidylserine, and other lipid groups.

Identifying lipid modifications suggests that they may serve as potential biomarkers for the early detection of atherosclerotic lesions.

Furthermore, this study indicates that analyzing GC could provide a more sensitive and reliable measure of cardiovascular risk than previously recognized.

## Figures and Tables

**Table 2:** Comparison of glycerophospholipids from subgroup-1 concentrations in the Patient and Control Groups.

Glycerophospholipids	Patient Group			Control Group			Fold-Change	*p*-Value	
	Median {*m*/*z*}	Lower Quartile	Upper Quartile	Median {*m*/*z*}	Lower Quartile	Upper Quartile		Raw	FDR *
Phosphatidylcholine (PC)									
PC (O-36:0/16:0)	181.8	150.8	245.2	134.3	101.8	167.5	1.35	0.006	0.0339
PC (O-36:5/20:5)	89.3	43.0	214.8	0.0	0.0	83.2	NA	0.016	0.0339
Phosphatidylethanolamine (PE)									
PE (36:1/16:0)	220.7	129.3	255.2	133.7	73.3	173.5	1.65	0.019	0.0339
PE (36:4/20:4)	833.7	572.3	935.2	571.0	448.8	749.7	1.46	0.048	0.0479
PE (38:3/16:0)	45.5	0.0	192.0	0.0	0.0	0.0	NA	0.033	0.0407
PE (38:4/16:0)	336.5	258.0	379.8	256.8	211.7	281.8	1.31	0.021	0.0339
PE (38:4/18:0)	1213.5	1005.8	1559.2	1003.8	789.0	1171.3	1.21	0.040	0.0441
PE (38:4/20:4)	3042.3	2741.7	3904.0	2524.3	1937.8	2890.2	1.21	0.008	0.0339
PE (38:5/20:4)	593.0	420.3	733.0	412.8	312.5	504.8	1.44	0.005	0.0339
PE (40:4/18:1)	0.0	0.0	1364.7	0.0	0.0	0.0	NA	0.018	0.0339
PE (40:5/18:1)	224.7	190.7	290.8	175.3	118.7	217.3	1.28	0.028	0.0382
PE (40:5/20:4)	265.8	198.5	336.3	180.2	87.2	212.3	1.48	0.005	0.0339
PE (O-38:5/20:4)	3455.2	2813.3	4838.8	2449.0	1509.7	3369.3	1.41	0.010	0.0339
PE (O-40:7/22:6)	309.7	253.5	412.3	127.3	0.0	330.7	2.43	0.014	0.0339
Phosphatidylglycerol (PG)									
PG (36:5/20:4)	793.2	702.3	1021.3	662.5	530.2	800.0	1.20	0.025	0.0359
PG (38:6/16:0)	621.5	499.5	1020.8	458.2	387.7	631.8	1.36	0.040	0.0441
PG (40:6/18:2)	131.3	0.0	445.8	0.0	0.0	0.0	NA	0.014	0.0339
PG (40:8/20:4)	797.0	626.5	1123.8	597.8	469.7	942.3	1.33	0.045	0.0473
Phosphatidylserine (PS)									
PS (36:1/18:1)	259.8	176.8	349.2	166.7	155.7	215.7	1.56	0.007	0.0339
PS (38:6/18:1)	249.2	177.7	266.8	153.8	31.0	248.0	1.62	0.033	0.0407
PS (O-36:1/18:0)	190.3	141.5	243.3	79.5	13.3	175.0	2.39	0.013	0.0339
PS (O-38:5/20:4)	353.7	287.0	427.3	230.7	150.3	338.2	1.53	0.022	0.0339
Lysophosphatidylcholine (LysoPC)									
No statistical significance observed									
Phosphatidic acid (PA)									
No statistical significance observed									
Phosphatidylinositol (PI)									
No statistical significance observed									
Phosphatidylinositol phosphate (PIP)									
No statistical significance observed									

* Adjusted *p*-value False Discovery Rate.

**Table 3:** Comparison of glycerophospholipid intensity from subgroup-2 in the Patient and Control Groups.

Glycerophospholipids	Patient Group			Control Group			Fold-Change	*p*-Value	
	Median {*m*/*z*}	Lower Quartile	Upper Quartile	Median {*m*/*z*}	Lower Quartile	Upper Quartile		Raw	FDR *
Cardiolipin (CL)									
CL (56:1/18:1)	56.7	29.8	105.5	110.7	85.3	147.0	0.51	0.001	0.0293
CL (68:3/18:1)	347.3	290.3	416.3	258.0	197.0	359.0	1.35	0.038	0.0485
CL (72:3/18:1)	402.7	352.7	494.0	291.7	268.7	388.3	1.38	0.017	0.0394
CL (72:8/20:4)	596.0	417.2	720.2	400.3	295.7	525.0	1.49	0.011	0.0387
CL (74:2/18:1)	323.5	250.7	472.8	267.8	195.0	282.7	1.21	0.045	0.0485
CL (76:10/20:4)	472.0	346.8	663.5	341.3	286.0	470.0	1.38	0.039	0.0485
CL (76:14/18:1)	345.2	275.8	398.7	267.7	224.0	326.7	1.29	0.028	0.0457
CL (76:7/20:4)	348.3	320.5	503.3	282.8	218.0	383.7	1.23	0.045	0.0485
CL (76:8/20:4)	971.3	831.0	1198.5	685.7	612.0	974.7	1.42	0.007	0.0385
CL (78:6/20:4)	793.2	702.3	1021.3	662.5	483.7	806.7	1.20	0.020	0.0413
CL (78:7/18:0)	1213.5	1005.8	1559.2	1003.8	750.3	1182.0	1.21	0.039	0.0485
CL (78:7/20:4)	3042.3	2741.7	3904.0	2524.3	1771.3	2828.3	1.21	0.005	0.0373
CL (78:9/20:4)	593.0	420.3	733.0	408.0	302.0	469.3	1.45	0.003	0.0293
CL (80:13/18:0)	242.8	181.8	283.5	194.8	109.3	215.0	1.25	0.020	0.0413
CL (80:9/20:4)	366.8	310.3	511.2	281.7	215.0	333.7	1.30	0.006	0.0385
CL (82:13/18:1)	259.8	176.8	349.2	166.7	152.0	211.7	1.56	0.003	0.0293
CL (82:4/18:1)	493.7	255.3	625.7	370.2	261.7	416.0	1.33	0.048	0.0485
CL (82:7/20:4)	376.0	328.3	447.5	320.0	229.3	361.3	1.18	0.015	0.0387
CL (82:9/20:4)	283.2	229.7	358.5	204.7	127.7	253.3	1.38	0.003	0.0293
CL (84:15/20:4)	196.2	142.3	241.7	144.7	123.3	185.0	1.36	0.048	0.0485
CL (88:11/16:0)	0.0	0.0	85.5	89.0	0.0	161.3	0.00	0.030	0.0457
CL (88:11/18:1)	0.0	0.0	190.3	262.3	0.0	529.0	0.00	0.015	0.0387
Diacylglycerolpyrophosphate (DGPP)									
DGPP (O-36:1/18:0)	358.7	276.0	467.7	290.7	195.7	345.7	1.23	0.032	0.0457
Dimethyl Phosphatidylethanolamine (DMPE)									
DMPE (24:5)	285.5	239.8	324.8	349.8	299.3	414.7	0.82	0.031	0.0457
DMPE (32:1/18:1)	485.3	346.8	624.2	346.7	301.0	489.7	1.40	0.045	0.0485
DMPE (34:1/18:0)	187.0	118.2	209.5	127.0	108.3	170.3	1.47	0.029	0.0457
DMPE (34:1/18:1)	1183.7	882.7	1380.2	994.2	856.0	1105.0	1.19	0.045	0.0485
DMPE (34:2/18:1)	538.2	415.8	627.0	365.2	326.7	454.0	1.47	0.015	0.0387
DMPE (34:4/20:4)	833.7	572.3	935.2	571.0	427.0	737.0	1.46	0.048	0.0485
DMPE (36:4/16:0)	336.5	258.0	379.8	256.8	211.3	286.0	1.31	0.024	0.0457
DMPE (38:3/16:0)	821.7	390.8	999.5	570.0	366.7	646.0	1.44	0.048	0.0485
DMPE (38:5/18:1)	224.7	190.7	290.8	175.5	145.0	214.0	1.28	0.016	0.0387
DMPE (38:6/20:4)	137.2	107.2	206.8	83.2	59.0	138.0	1.65	0.013	0.0387
DMPE (40:7/20:4)	3202.3	2312.0	4661.7	2404.2	1613.7	3542.0	1.33	0.048	0.0485
DMPE (42:8/20:4)	1125.2	384.3	1410.3	465.0	248.7	859.3	2.42	0.031	0.0457
DMPE (42:8/22:6)	208.3	156.8	333.3	141.2	109.3	211.7	1.48	0.016	0.0387
Monomethyl Phosphatidylethanolamine (MMPE)									
MMPE (34:5/20:4)	2529.0	1809.3	2991.7	1651.2	1286.0	2259.3	1.53	0.009	0.0385
MMPE (36:5/20:4)	3861.8	3311.0	4983.8	2706.8	2312.0	3798.0	1.43	0.009	0.0385
MMPE (38:7/22:6)	413.5	339.8	508.5	331.0	219.0	377.0	1.25	0.026	0.0457
Phosphoethanolamine (NAPE)									
No statistical significance observed									
Sphingolipids (SM)									
SM 36:2;O4	368.7	354.0	403.2	329.0	303.7	374.0	1.12	0.030	
SM 38:2;O4	270.8	228.2	333.2	233.2	178.3	271.5	1.16	0.033	

* Adjusted *p*-value False Discovery Rate.

**Table 4:** Results of the multivariate logistic regression performed on statistically significant variables.

Name of Compounds	Odds Ratio (95% CI)	*p*-Value	Odds Ratio (95% CI)	*p*-Value
Separate models for each class			General model for all classes *	
Glycerophospholipids Sg-1. AUC (95% CI): 0.942 (0.876–0.999)				
PC 36:0/16:0 (unit 10)	1.246 (1.042–1.490)	0.0157	1.293 (1.027–1.627)	0.0286
PE O-40:7/22:6 (unit 10)	1.119 (1.039–1.205)	0.0028	1.083 (1.003–1.169)	0.0429
PG 40:8/20:4 (unit 10)	1.053 (1.008–1.101)	0.0219	-	
Glycerophospholipids Sg-2. AUC (95% CI): 0.942 (0.865–0.999)				
DMPE 42:8/22:6 (unit 10)	1.198 (1.020–1.406)	0.0276	1.215 (1.003–1.473)	0.0468
CL 56:1/18:1 (unit 10)	0.748 (0.567–0.988)	0.0406	0.702 (0.503–0.978)	0.0366
CL 82:13/18:1 (unit 10)	1.264 (1.017–1.572)	0.0350	-	
Sphingolipids AUC (95% CI): 0.712 (0.548–0.877)				
SM 38:2;O4 (unit 10)	1.146 (1.016–1.291)	0.0260	-	

* AUC (95% CI): 0.958 (0.904–0.999).

## Data Availability

All data underlying the results are available within the article and its Supplementary Materials.

## Supplementary Materials

The following supporting information can be downloaded at https://www.mdpi.com/article/10.3390/biomedicines13030643/s1, Table S1: Lipid compounds analysed in this study.

## References

[1] Beyene H.B., Giles C., Huynh K., Wang T., Cinel M., Mellett N.A., Olshansky G., Meikle T.G., Watts G.F., Hung J. (2023). Metabolic phenotyping of BMI to characterize cardiometabolic risk: Evidence from large population-based cohorts. Nat. Commun..

[2] Meikle P.J., Wong G., Barlow C.K., Kingwell B.A. (2014). Lipidomics: Potential role in risk prediction and therapeutic monitoring for diabetes and cardiovascular disease. Pharmacol. Ther..

[3] Gu P.S., Su K.W., Yeh K.W., Huang J.L., Lo F.S., Chiu C.Y. (2023). Metabolomics Analysis Reveals Molecular Signatures of Metabolic Complexity in Children with Hypercholesterolemia. Nutrients.

[4] Dobrowolski P., Kabat M., Kępka C., Januszewicz A., Prejbisz A. (2022). Atherosclerotic cardiovascular disease burden in patients with familial hypercholesterolemia: Interpretation of data on involvement of different vascular beds. Pol. Arch. Intern. Med..

[5] Nurmohamed N.S., Kraaijenhof J.M., Mayr M., Nicholls S.J., Koenig W., Catapano A.L., Stroes E.S.G. (2023). Proteomics and lipidomics in atherosclerotic cardiovascular disease risk prediction. Eur. Heart J..

[6] Duan Y., Gong K., Xu S., Zhang F., Meng X., Han J. (2022). Regulation of cholesterol homeostasis in health and diseases: From mechanisms to targeted therapeutics. Signal Transduct. Target. Ther..

[7] Jayawardana K.S., Mundra P.A., Giles C., Barlow C.K., Nestel P.J., Barnes E.H., Kirby A., Thompson P., Sullivan D.R., Alshehry Z.H. (2019). Changes in plasma lipids predict pravastatin efficacy in secondary prevention. JCI Insight.

[8] Mundra P.A., Barlow C.K., Nestel P.J., Barnes E.H., Kirby A., Thompson P., Sullivan D.R., Alshehry Z.H., Mellett N.A., Huynh K. (2018). Large-scale plasma lipidomic profiling identifies lipids that predict cardiovascular events in secondary prevention. JCI Insight.

[9] Fahy E., Cotter D., Sud M., Subramaniam S. (2011). Lipid classification, structures and tools. Biochim. Biophys. Acta.

[10] Gibellini F., Smith T.K. (2010). The Kennedy pathway—De novo synthesis of phosphatidylethanolamine and phosphatidylcholine. IUBMB Life.

[11] Patel D., Witt S.N. (2017). Ethanolamine and Phosphatidylethanolamine: Partners in Health and Disease. Oxid. Med. Cell. Longev..

[12] Vance D.E. (2008). Role of phosphatidylcholine biosynthesis in the regulation of lipoprotein homeostasis. Curr. Opin. Lipidol..

[13] Avela H.F., Sirén H. (2020). Advances in lipidomics. Clin. Chim. Acta.

[14] Visseren F.L.J., Mach F., Smulders Y.M., Carballo D., Koskinas K.C., Bäck M., Benetos A., Biffi A., Boavida J.M., Capodanno D. (2021). 2021 ESC Guidelines on cardiovascular disease prevention in clinical practice. Eur. Heart J..

[15] Ryan M.J., Grant-St James A., Lawler N.G., Fear M.W., Raby E., Wood F.M., Maker G.L., Wist J., Holmes E., Nicholson J.K. (2023). Comprehensive Lipidomic Workflow for Multicohort Population Phenotyping Using Stable Isotope Dilution Targeted Liquid Chromatography-Mass Spectrometry. J. Proteome Res..

[16] Zivkovic A.M., Wiest M.M., Nguyen U.T., Davis R., Watkins S.M., German J.B. (2009). Effects of sample handling and storage on quantitative lipid analysis in human serum. Metabolomics.

[17] Lin D.Y. (2019). A simple and accurate method to determine genomewide significance for association tests in sequencing studies. Genet. Epidemiol..

[18] Arnett D.K., Blumenthal R.S., Albert M.A., Buroker A.B., Goldberger Z.D., Hahn E.J., Himmelfarb C.D., Khera A., Lloyd-Jones D., McEvoy J.W. (2019). 2019 ACC/AHA Guideline on the Primary Prevention of Cardiovascular Disease: A Report of the American College of Cardiology/American Heart Association Task Force on Clinical Practice Guidelines. Circulation.

[19] Wang J., Xu J., Liu T., Yu C., Xu F., Wang G., Li S., Dai X. (2024). Biomechanics-mediated endocytosis in atherosclerosis. Front. Cardiovasc. Med..

[20] Sánchez-Vinces S., Garcia P.H.D., Silva A.A.R., Fernandes A.M.A.P., Barreto J.A., Duarte G.H.B., Antonio M.A., Birbrair A., Porcari A.M., Carvalho P.O. (2023). Mass-Spectrometry-Based Lipidomics Discriminates Specific Changes in Lipid Classes in Healthy and Dyslipidemic Adults. Metabolites.

[21] Züllig T., Köfeler H.C. (2021). High resolution mass spectrometry in lipidomics. Mass Spectrom. Rev..

[22] Köfeler H.C., Ahrends R., Baker E.S., Ekroos K., Han X., Hoffmann N., Holčapek M., Wenk M.R., Liebisch G. (2021). Recommendations for good practice in MS-based lipidomics. J. Lipid Res..

[23] Hishikawa D., Hashidate T., Shimizu T., Shindou H. (2014). Diversity and function of membrane glycerophospholipids generated by the remodeling pathway in mammalian cells. J. Lipid Res..

[24] Meikle P.J., Wong G., Tsorotes D., Barlow C.K., Weir J.M., Christopher M.J., MacIntosh G.L., Goudey B., Stern L., Kowalczyk A. (2011). Plasma lipidomic analysis of stable and unstable coronary artery disease. Arterioscler. Thromb. Vasc. Biol..

[25] Ekroos K., Jänis M., Tarasov K., Hurme R., Laaksonen R. (2010). Lipidomics: A tool for studies of atherosclerosis. Curr. Atheroscler. Rep..

[26] Zhang Z., Karu N., Kindt A., Singh M., Lamont L., van Gammeren A.J., Ermens A.A.M., Harms A.C., Portengen L., Vermeulen R.C.H. (2024). Association of Altered Plasma Lipidome with Disease Severity in COVID-19 Patients. Biomolecules.

[27] Stegemann C., Pechlaner R., Willeit P., Langley S.R., Mangino M., Mayr U., Menni C., Moayyeri A., Santer P., Rungger G. (2014). Lipidomics profiling and risk of cardiovascular disease in the prospective population-based Bruneck study. Circulation.

[28] Eggers L.F., Schwudke D. (2018). Shotgun Lipidomics Approach for Clinical Samples. Methods Mol. Biol..

[29] Meikle T.G., Huynh K., Giles C., Meikle P.J. (2021). Clinical lipidomics: Realizing the potential of lipid profiling. J. Lipid Res..

[30] Gencer B., Morrow D.A., Braunwald E., Goodrich E.L., Hilvo M., Kauhanen D., Sabatine M.S., Laaksonen R., O’Donoghue M.L. (2022). Plasma ceramide and phospholipid-based risk score and the risk of cardiovascular death in patients after acute coronary syndrome. Eur. J. Prev. Cardiol..

[31] Laaksonen R., Ekroos K., Sysi-Aho M., Hilvo M., Vihervaara T., Kauhanen D., Suoniemi M., Hurme R., März W., Scharnagl H. (2016). Plasma ceramides predict cardiovascular death in patients with stable coronary artery disease and acute coronary syndromes beyond LDL-cholesterol. Eur. Heart J..

[32] Timmerman N., Waissi F., Dekker M., de Borst G.J., van Bennekom J., de Winter R.J., Hilvo M., Jylhä A., Pasterkamp G., de Kleijn D.P.V. (2022). Ceramides and phospholipids in plasma extracellular vesicles are associated with high risk of major cardiovascular events after carotid endarterectomy. Sci. Rep..

[33] Dobrowolski P., Prejbisz A., Kuryłowicz A., Baska A., Burchardt P., Chlebus K., Dzida G., Jankowski P., Jaroszewicz J., Jaworski P. (2022). Metabolic syndrome—A new definition and management guidelines: A joint position paper by the Polish Society of Hypertension, Polish Society for the Treatment of Obesity, Polish Lipid Association, Polish Association for Study of Liver, Polish Society of Family Medicine, Polish Society of Lifestyle Medicine, Division of Prevention and Epidemiology Polish Cardiac Society, “Club 30” Polish Cardiac Society, and Division of Metabolic and Bariatric Surgery Society of Polish Surgeons. Arch. Med. Sci..

