# Supplementary material for: Early Changes in the Plasma Lipidome of People at Very High Cardiovascular Risk: A New Approach to Assessing the Risk of Cardiovascular Changes
Source: Biomedicines. 2025 Mar 6;13(3):643. doi: 10.3390/biomedicines13030643 (PMC11940131; doi:10.3390/biomedicines13030643)
Supplement: Supplementary file 1 [file biomedicines-13-00643-s001.zip › biomedicines-3466479-supplementary.pdf]

Supplementary Table S1 Lipid compounds analysed in this study.

**Glycerophospholipids (GC)**			
**Glycerophospholipids (subgroup-1) n = 222***	**Glycerophospholipids (subgroup-2) n = 202***	**Sphingolipids n = 20***	**Fatty Acyls n = 11***
PA – Phosphatidic acid	Cl – Cardiolipin	SM – Sphingomyelin	FFA – Free Fatty Acid
PC – Phosphatidylcholine	DGPP – Diacylglycerolpyrophosphate	Cer – Ceramide	ME – Methyl Ester
PE – Phosphatidyletanolamine	MMPE Monomethyl phosphatidylethanolamine	HexCer – Hexosylceramide	EE - Ethyl Ester
PG – Phosphatidylglycerol	DMPE – Dimethyl phosphatidylethanolamine	Hex2Cer – Dihexosylceramide	GlcdE – Glycidol Ester
PI – Phosphatidylinositol	PIP – Phosphatidylinositol Phosphatate	SgalCer – Sulphatides	OAHFA_16:0/ -O-16:0 Hydroxy Fatty Acid
PS – Phosphatidylserine	PIP2 – Phosphatidylinositol Biphosphatate	CerP – Ceramide-phosphatate	OAHFA_16:1/ -O-16:1 Hydroxy Fatty Acid
	PIP3 – PhosphatidylinositolTriphosphatate	CerPE – Ceramide phosphoethanolamine	OAHFA_18:0/ -O-18:0 Hydroxy Fatty Acid
	CDPDAG – 3-Citidine Diphosphatate Diacylglycerol	IPC – Inositophosphorylceramide	OAHFA_18:1/ -O-18:1 Hydroxy Fatty Acid
	NAPE – N-acyl phosphoethanolamine	MIPC – Mannosyl-inositolPceramide	OAHFA_18:2/ -O-18:2 Hydroxy Fatty Acid
		M(IP)2C – Mannosyl-diinositolPceramide	
		Hex3Cer – Trihexosylceramide	
		GM3 – Monosialoganglioside	
		GM2 – MonosialogangliosideGalNAc	
		GM1– MonosialogangliosideGalGalNAc	
		GD3 – Disialoganglioside	
		GD2 – DisialogangliosideGalNAc	

	GD1 – DisialogangliosideGalGalNAc	
	GT3 – Trisialoganglioside	

*Total number of tested compounds. Details with change values are presented in Tables 2-4.
